# Predictive value of abdominal wall scar score for pelvic floor function rehabilitation, vaginal microecology and complications after cesarean section

**DOI:** 10.7717/peerj.16012

**Published:** 2023-09-15

**Authors:** Yanhong Yang, Hailan Yang, Jingru Ji, Ye Zhao, Yinfang He, Junyan Wu

**Affiliations:** First Hospital of Shanxi Medical University, Taiyuan, China

**Keywords:** Abdominal wall scar score, After cesarean section again, Rehabilitation of pelvic floor function, Vaginal microecology, Complications, Predictive value

## Abstract

**Objective:**

To explore the predictive value of the abdominal wall scar score for pelvic floor function rehabilitation, vaginal microecology and complications after cesarean section.

**Methods:**

A total of 120 pregnant women who underwent cesarean section in our hospital from January to December 2022 were selected. The patients were divided into observation group (score ≥ 60, *n* = 52) and control group (score < 60, *n* = 68) according to the preoperative score of abdominal wall scar and whether the score exceeded 60. The pelvic floor function rehabilitation, vaginal microecology and complications were compared between the two groups, and the score of abdominal wall scar was evaluated by receiver operating characteristic (ROC) curve. The predictive value of pelvic floor function rehabilitation, vaginal microecology and complications after cesarean section was evaluated.

**Results:**

There were significant differences between the two groups in postpartum class I and class II muscle fiber strength and pelvic floor muscle potential (*P* < 0.05). ROC curve showed that the AUC of abdominal scar score in predicting pelvic floor function rehabilitation was 0.806 (95% CI [0.684–0.927]), the specificity was 80.17%, and the sensitivity was 79.76%. There was significant difference in the abnormal rate of leukocte estrase (LE) and Acetylaminoglucosidase (NAG) between the two groups (*P* < 0.05). ROC curve showed that the AUC of abdominal scar score in predicting vaginal microecology was 0.871 (95% CI [0.776–0.966]), the specificity was 85.09%, and the sensitivity was 82.36%. There was significant difference in the incidence of postpartum complications between the two groups (*P* < 0.05). ROC curve showed that the AUC of abdominal scar score in predicting complications was 0.844 (95% CI [0.735–0.953]), the specificity was 82.27%, and the sensitivity was 81.15%.

**Conclusion:**

The abdominal scar score has a certain effect on predicting the recovery of pelvic floor function, vaginal microecology and complications after cesarean section. Therefore, it can help the medical staff to adjust the treatment measures in time, which can be used as a means of preoperative auxiliary examination.

## Introduction

Cesarean section is an important operation in obstetrics. The clinical indications for cesarean section are strictly controlled. With the improvement of assisted reproductive technology, the cesarean section rate is also increasing. In addition, with the release of the second child policy, the rate of second pregnancy in scar uterus has an increasing trend (Sholapurkar, 2018). Most studies have shown that scar uterus has more postoperative complications (Tekelioğlu et al., 2021). Assisted reproductive technology refers to the use of medical technology to assist in achieving pregnancy and childbirth. It includes many different technologies, such as external fertilization (IVF), embryo transfer, surrogacy, *etc*. The development of these technologies provides an opportunity for couples who are unable to conceive or become pregnant naturally due to physiological factors, allowing them to achieve their desire to have children. The second child policy usually refers to the regulation of the national family planning policy, allowing couples to have a second child. In the past, many countries implemented strict family planning policies, such as China’s one child policy. However, with changes in population structure and socio-economic development, some countries have begun to adjust their policies and relax restrictions on second children. Due to the development of assisted reproductive technology, many couples can use these technologies to achieve their desire to have multiple children, even if they may have difficulty giving birth. Under the policy of allowing for the birth of a second child, some couples may choose to use assisted reproductive technology to achieve their fertility plan. In recent years, studies have shown that the incidence of uterine rupture and massive bleeding increases gradually when scar uterus is delivered again, while at present, there are still more special parturients who choose cesarean section (Tekiner et al., 2018).

Pelvic floor dysfunction (PFD) is a common gynecological disease, which includes pelvic organ prolapse, urinary incontinence, fecal incontinence, *etc*. in clinical practice, of which urinary incontinence is the most common, which causes some damage to the patient’s pelvic floor muscles during pregnancy and delivery, perineal nerve damage can lead to local muscle atrophy, and then aggravate the damage to pelvic muscles (Zachovajeviene et al., 2019), followed by perineal tear, fetal head circumference, fetal body mass. The second stage of labor, the number of deliveries, and high-risk pregnancies can all increase damage to the pelvic floor muscles, leading to PFD (Navarro-Brazález et al., 2020). A study (Wu et al., 2021) found that prenatal and prenatal overweight, as well as a history of induced labor, can all affect postpartum pelvic floor muscle strength, leading to poor pelvic floor muscle function in postpartum women from 6 to 8 weeks. It is recommended that women participate in pelvic floor function screening in the early postpartum period, and pregnant women with abnormal pelvic floor muscle strength receive pelvic floor rehabilitation training to prevent PFD disease (Verbeek & Hayward, 2019). Pelvic floor dysfunction refers to abnormal symptoms such as stress urinary incontinence and pelvic organ prolapse caused by abnormal pelvic floor support structure and injury (Schütze et al., 2022). Clinical diagnosis of abnormal changes in pelvic floor function often relies on imaging methods. Pelvic ultrasound has the advantages of simple, noninvasive and real-time (Wilson et al., 2021). Specifically, pelvic floor ultrasound is a simple and non-invasive imaging technique that allows for direct observation and evaluation of pelvic floor structure and function without the need for traumatic examination. Pelvic floor ultrasound can provide real-time imaging, allowing doctors to directly observe and evaluate the dynamic changes in pelvic floor structure and function. This enables doctors to more accurately grasp the patient’s pelvic floor condition and promptly identify potential problems. Pelvic floor ultrasound provides objective and accurate information by directly observing and measuring the structure and function of the pelvic floor. This helps doctors better understand the patient’s pelvic floor condition in order to develop personalized intervention plans. By evaluating the structure and function of the pelvic floor, doctors can develop personalized intervention plans based on the specific situation of each postpartum woman. For example, if pelvic floor muscle damage or functional abnormalities are found, targeted rehabilitation training or other treatment methods can be carried out to improve pelvic floor function and recovery. The postoperative complications of pregnant women with scar uterus need to be paid enough attention, and the pelvic floor structure and function of these women should be accurately evaluated, it is conducive to the clinical development of individualized intervention program, which is of great significance to improve the postpartum recovery of puerpera. A large number of studies have confirmed that the composition of vaginal microbial community is diverse. After the vaginal microecological balance is broken, the ability to resist pathogen infection and colonization is reduced, which can mediate inflammation and infection (Ekin et al., 2018). However, there is no preoperative technique that can effectively predict pelvic floor function rehabilitation, vaginal microecology and complications, previous studies have found that there is a certain correlation between abdominal wall scar and pelvic adhesion in pregnant women with cesarean section again (Carrière et al., 2023). The abdominal wall scar score can provide relevant information about abdominal wall incisions, which is related to the supporting structure of the pelvic floor to a certain extent. The evaluation of abdominal wall scar can be used as an indicator of vaginal microecological disorder after cesarean section. Poor scar healing or infection may affect the vaginal microecological environment. Abdominal scar score can reflect the healing of abdominal incision after caesarean section and the overall surgical repair. Therefore, this article aims to study the predictive value of abdominal scar score for pelvic floor function rehabilitation, vaginal microecology and complications after cesarean section again, and provide the basis for clinical treatment, assist doctors and patients in developing more effective personalized rehabilitation plans and taking corresponding preventive measures to improve rehabilitation outcomes and reduce the risk of complications.

## Materials and Methods

### Materials

Study subjects 120 pregnant women who underwent cesarean section in our hospital from January to December 2022 were selected. This study were approved by the ethics committee of the First Hospital of Shanxi Medical University and abided by the ethical guidelines of the Declaration of Helsinki and also agreed to waive informed consent. Inclusion criteria: (1) gestational age was 37–42 weeks; (2) those with only one previous cesarean section and more than 2 years from the last cesarean section; (3) there were no complications of pregnancy such as anemia and gestational diabetes mellitus; (4) no history of other types of abdominal surgery; (5) the patient was mentally fit and could cooperate with the researchers to complete the investigation. Exclusion criteria: (1) placenta previa and implantation; (2) the pregnant woman herself is of scar constitution; (3) hematoma, poor wound healing or postoperative fever after the previous cesarean section; (4) the incision of uterine serosa and peritoneum failed to be effectively sutured after operation; (5) patients with severe organic organ disease or other contraindications to surgery; (6) poor compliance and who were unable to cooperate to complete the survey. All cesarean sections were performed by the same attending gynecologist, according to whether the score exceeded 60, the patients were divided into observation group (score ≥ 60, *n* = 52) and control group (score < 60, *n* = 68). There was no significant difference between the two groups in age, gestational weeks, height, weight, number of pregnancies, operation time and other general data (*P* > 0.05), which was comparable, see Table 1.

**Table 1:** Comparison of general data of 120 pregnant women who underwent cesarean section again from January to December 2022.

Variable	Control group (*n* = 68)	Observation group (*n* = 52)	*t-values*	*P*
Age (years)	28.16 ± 2.38	28.28 ± 2.42	0.272	0.786
Gestational weeks (weeks)	38.87 ± 1.39	39.15 ± 1.28	1.131	0.260
Height (cm)	159.43 ± 7.12	158.43 ± 7.21	0.758	0.450
Weight (kg)	58.32 ± 6.32	56.12 ± 6.73	1.837	0.069
Number of pregnancies	2.55 ± 0.37	2.50 ± 0.31	0.786	0.433
Operation time (min)	84.26 ± 26.35	86.58 ± 28.01	0.465	0.643

### Method

#### Abdominal scar score

The condition of abdominal wall scar was evaluated by POSAS scale (Schütze et al., 2022), Including vascular distribution, color, thickness, surface roughness, softness and surface area, when filling in, three physicians should score independently and take the average value; pain, pruritus, color, thickness, softness and self perception. The score of each parameter is from 1 to 10, which represents from normal skin to the worst case; the lowest overall score is 12 points, and the highest is 120 points. The lower the score, the better the condition of scar skin. To ensure its reliability and effectiveness, consistency testing is the first step, which involves multiple evaluators evaluating the same scar and calculating the consistency between evaluators. Secondly, effectiveness testing, which evaluates whether the scoring system can accurately evaluate different types of scars.

#### Observation indexes

(1) Pelvic floor function rehabilitation: the researchers are responsible for evaluating pelvic floor muscle strength, pelvic floor muscle potential and pelvic floor dysfunction scores after delivery. The pelvic floor muscle strength was measured by the Melander biological stimulation feedback instrument (Shanghai, China), the pelvic floor muscle fiber strength and pelvic floor muscle potential of type I and II puerpera were measured, according to the perineal muscle strength test method, the muscle strength was graded (grade 0–V). The higher the grade, the greater the maternal pelvic floor muscle strength. Muscle potential is used to evaluate the electrophysiological changes produced by muscle excitation, mainly reflecting muscle tension and muscle fatigue. The greater the measured value, the greater the pelvic floor muscle strength and the weaker the fatigue degree. The Chinese version of female pelvic floor dysfunction questionnaire was used to evaluate pelvic floor function. There are 20 items in total, including three dimensions that affect daily life: bladder (six items), intestine (eight items) and pelvic cavity (six items). The score of each item is 0–4 points. The average score of each dimension multiplied by 25 is the score of this dimension (100 points). The total score is 300 points. The higher the score, the more severe the pelvic floor dysfunction of the puerpera. (2) Vaginal microecology: the vaginal microecological index was detected after delivery. The examinee took the bladder lithotomy position, Expose the cervix, disinfect the vulva, and use two sterile cotton swabs to take 1/3 of the secretion on the lateral wall of the vagina for examination. The vaginal microecological detector of bd-500 (Chongqing, China) and its supporting kit were used to detect pH, catalase (H_2_O_2_), leukocyte esterase (LE), sialyl glycosidase (SNA) β—Glucuronidase (β-GD), acetylglucosaminidase (NAG) levels. Each index is divided into normal and abnormal, in which the normal range of pH value is 3.8~4.5, and it is judged as abnormal if it exceeds the range, if other indicators are tested positive, it is judged as abnormal. (3) Complications: the recent postpartum complications of the two groups were compared, including postpartum hemorrhage, puerperal infection, abdominal incision infection.

### Statistical analysis

SPSS 21.0 was used for analysis. The measurement data in accordance with normal distribution in the experimental data were expressed by ${\mathrm{\bar X}}$ ± S, The two groups were compared by group t-test, The counting data were expressed by the number of cases or rate, and the two groups were compared using χ^2^ inspection, Kruskal Wallis rank sum test was used to compare the classification data of multiple groups, and receiver operating characteristic (ROC) curve was used to evaluate the predictive value of related factors, with *P* < 0.05 as the difference statistically significant.

## Results

### Comparison of postpartum class I and II muscle fiber strength grade and pelvic floor muscle potential between the two groups of pregnant women

There were statistically significant differences in the strength grade of class I and II muscle fibers and pelvic floor muscle potential between the two groups after delivery (*P* < 0.05). See Table 2.

**Table 2:** Comparison of postpartum class I and II muscle fiber strength grade and pelvic floor muscle potential between the two groups of pregnant women.

Project	Control group (*n* = 68)	Observation group (*n* = 52)	*t-values*	*P*
Class I muscle fiber strength grade				
0 Level	18	3	20.735	0.000
I Grade	16	6		
II Grade	12	7		
III Grade	9	8		
IV Grade	7	12		
V Grade	6	16		
Class II muscle fiber strength grade				
0 Level	14	1	20.995	0.000
I Grade	15	4		
II Grade	10	5		
III Grade	11	11		
IV Grade	8	14		
V Grade	10	17		
Pelvic floor muscle potential	20.03 ± 2.28	24.54 ± 2.36	10.576	0.000
Pelvic floor dysfunction score	42.35 ± 4.46	34.64 ± 4.22	9.604	0.000

### Predictive value of abdominal scar score for pelvic floor functional rehabilitation

**Figure 1:**
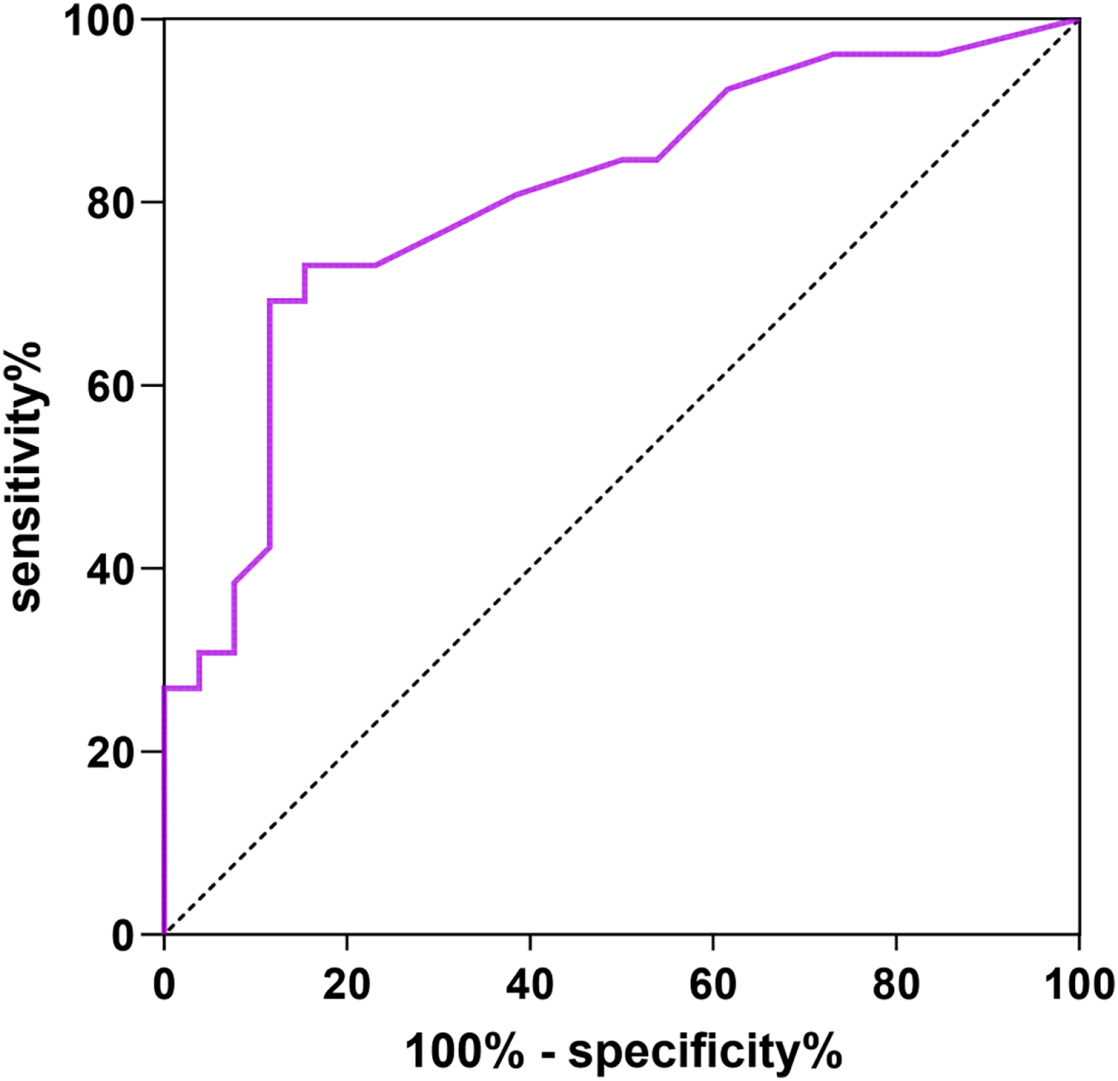
ROC curve of abdominal scar score predicting pelvic floor function rehabilitation.

Abdominal scar score was used as the test variable, pelvic floor dysfunction as the dependent variable (1 = yes, 0 = no). The ROC curve showed that the AUC of abdominal scar score for pelvic floor function rehabilitation was 0.806 (95% CI [0.684–0.927]). The specificity was 80.17% and the sensitivity was 79.76%. See Fig. 1 for details.

### Comparison of postpartum vaginal microecology between the two groups of pregnant women

There was a statistically significant difference in the abnormal rates of leukocte estrase (LE) and Acetylaminoglucosidase (NAG) between the two groups after delivery (*P* < 0.05). See Table 3.

**Table 3:** Comparison of postpartum vaginal microecology between the two groups of pregnant women.

Index	Control group (*n* = 68)	Observation group (*n* = 52)	*t-values*	*P*
pH				
Normal	21	15	0.058	0.809
Abnormal	47	37		
H_2_O_2_				
Normal	13	14	1.030	0.310
Abnormal	55	38		
LE				
Normal	54	22	17.471	<0.001
Abnormal	14	30		
SNA				
Normal	56	35	3.640	0.056
Abnormal	12	17		
β-GD				
Normal	66	50	0.075	0.784
Abnormal	2	2		
NAG				
Normal	53	31	4.712	0.030
Abnormal	15	21		

### Predictive value of abdominal scar score on vaginal microecology

**Figure 2:**
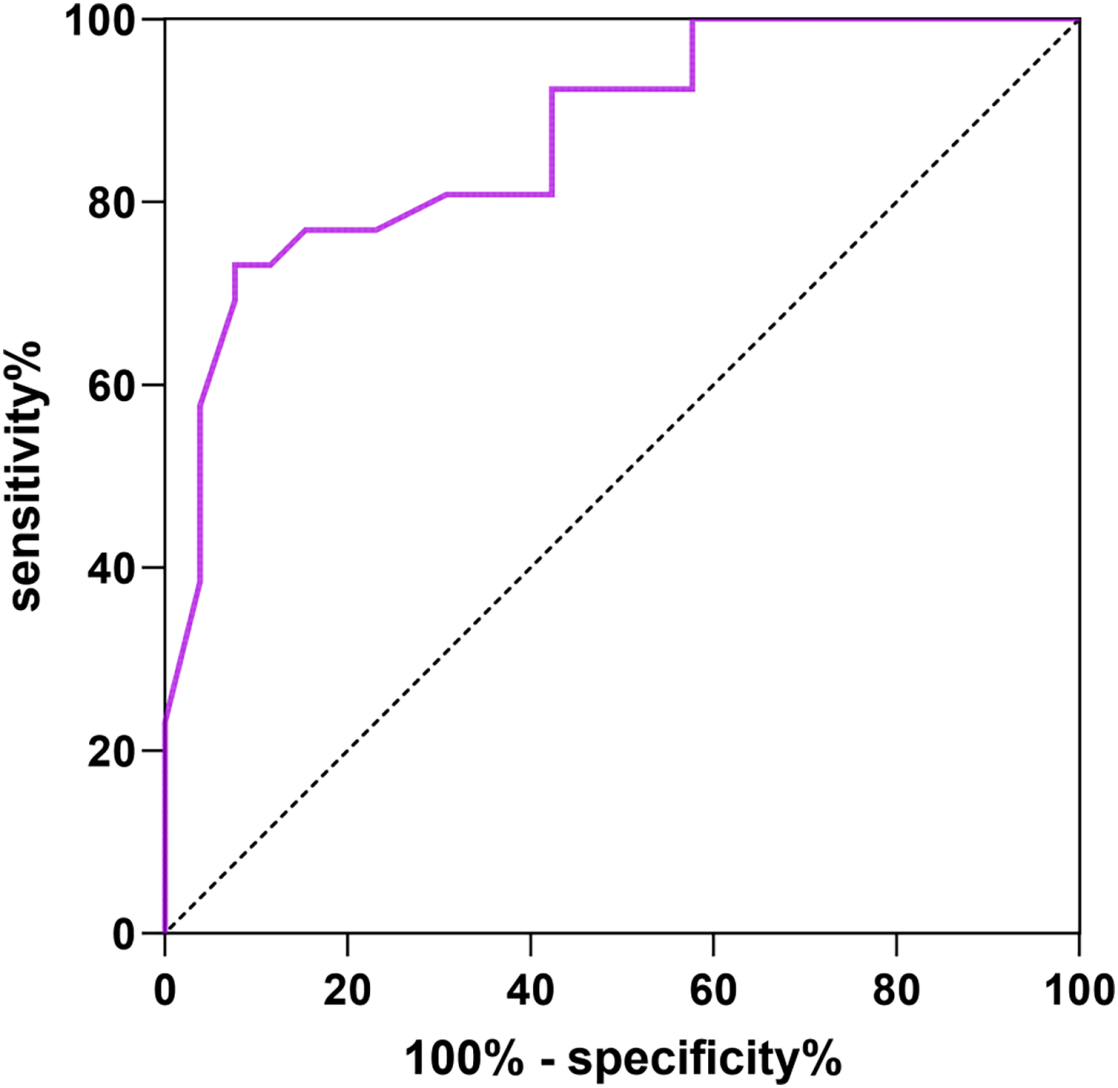
ROC curve of abdominal scar score predicting vaginal microecological abnormalities.

The abdominal scar score was taken as the test variable, and the vaginal microecological abnormality was taken as the dependent variable (1 = yes, 0 = no), the ROC curve showed that the AUC of abdominal scar score for vaginal microecology prediction was 0.871 (95% CI [0.776–0.966]), the specificity was 85.09% and the sensitivity was 82.36%. See Fig. 2 for details.

### Comparison of recent postpartum complications between the two groups of pregnant women

The difference in the incidence of recent postpartum complications between the two groups was statistically significant (*P* < 0.05). See Table 4.

**Table 4:** Comparison of recent postpartum complications between the two groups of pregnant women.

Complication	Control group (*n* = 68)	Observation group (*n* = 52)	χ^2^	*P*
Postpartum hemorrhage	2	1		
Puerperal infection	1	1		
Abdominal incision infection	4	0		
Total incidence (%)	10 (14.71)	1 (1.92)	4.784	0.029

### Predictive value of abdominal scar score for complications

**Figure 3:**
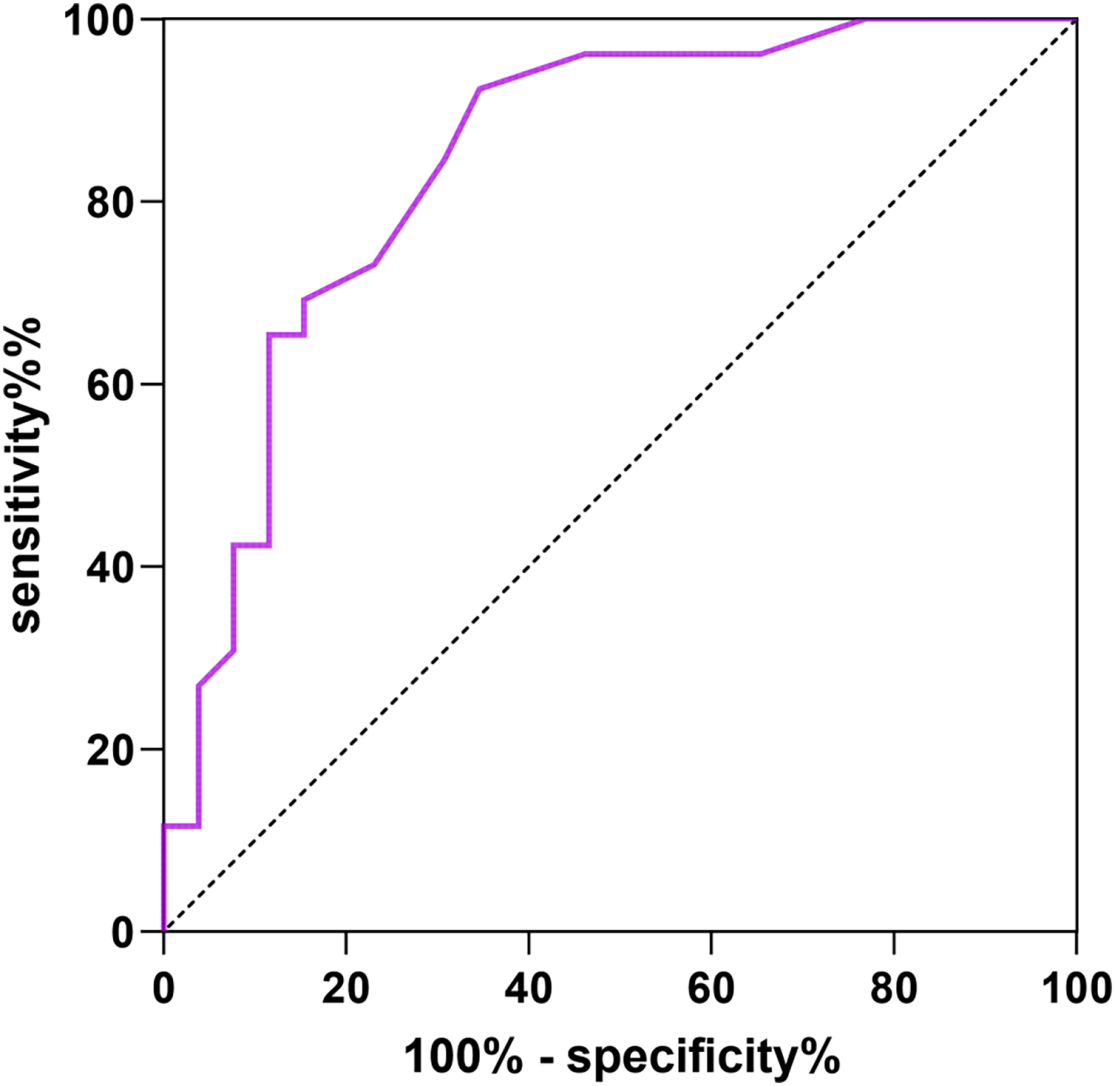
ROC curve of abdominal scar score for predicting complications.

The ROC curve was drawn with abdominal scar score as the test variable and complications as the dependent variable (1 = yes, 0 = no), The AUC of abdominal scar score for predicting complications was 0.844 (95% CI [0.735–0.953]), The specificity was 82.27% and the sensitivity was 81.15%. See Fig. 3 for details.

## Discussion

The reoperation of scar uterus is easy to cause many complications, which can be divided into short-term complications and long-term complications. The short-term complications are: postpartum bleeding, infection, amniotic fluid embolism, organ damage, thrombosis, *etc*. Long term complications mainly include endometriosis, pelvic adhesion, incision diverticulum, incision pregnancy, placenta praevia, uterine rupture, *etc*. After cesarean section, pregnant women leave scars on the abdominal wall. Previous studies have shown that for pregnant women with scarred uterus, although cesarean section can avoid uterine rupture during delivery, it also increases the risk of surgical complications (Hanacek et al., 2020). Relevant studies have found that the probability of neonatal asphyxia, infection, and other adverse outcomes of cesarean section in pregnant women with scarred uterus has increased, it is suggested that the abdominal wall scar itself has a certain impact on the outcome of cesarean section (Eleje et al., 2023; Abdelazim et al., 2019). At the same time, domestic research in recent years believes that pregnant women with scarred uterus are pulled by the incision brought by previous surgery. The incidence of postpartum hemorrhage was significantly higher than that of pregnant women in the control group (Qiu et al., 2022). The above studies all suggest that abdominal wall scar has a certain impact on the prognosis of cesarean section pregnant women.

Abnormal pelvic floor muscle strength is the primary manifestation of pelvic floor dysfunction. When evaluating pelvic floor function, the muscle strength of class I and II muscle fibers can be measured (Zhu et al., 2022). Transperineal pelvic floor ultrasound imaging technology can observe the supporting structure of pelvic floor from any angle, and the visual field plane can rotate and translate, and the observed tissue structure relationship is clearer. The damage of pelvic floor muscles after cesarean section in the second pregnancy of scar uterus with a history of cesarean section is greater than that of a single pregnancy, this damage is both direct mechanical damage and indirect damage to pelvic muscles caused by pudendal nerve damage (Ptak et al., 2019). The study found that compared with the maximum skeletal muscle extension rate of non pregnant women, the stretching rate of pelvic floor muscles of pregnant women with scar uterus who have a history of cesarean section was significantly increased during delivery, and the higher the stretching force of levator ani muscle, Therefore, the excessive stretching of pelvic floor muscles, soft birth canal, fascia and ligaments is more likely to lead to abnormal vaginal pressure and pelvic floor muscle strength (Wang et al., 2021). In the results of this study, there were significant differences in the strength grade of class I and II muscle fibers and pelvic floor muscle potential between the two groups after delivery, suggesting that the abdominal wall scar score is related to the recovery of pelvic floor function. LE is one of the specific enzymes contained in leukocytes in the body, which can reflect the level of leukocytes in vaginal secretions, however, because LE only exists in the neutrophils of inflammatory cells, abnormal Le indicates inflammation in female vagina (Song et al., 2021). Elevated NAG activity is common in tubulointerstitial disease, congenital tubulopathy, and glomerulonephritis, however, abnormal NAG in vaginal secretions is mostly seen in the excessive bacterial content in the vagina, which can be considered as bacterial vaginosis, and the risk of cervical infection in patients is also high (Martín-Peláez et al., 2022). In the results of this study, there was a significant difference in the abnormal rates of LE and NAG between the two groups after delivery, suggesting that the abdominal scar score can indicate vaginal local infection and inflammation. In the results of this study, there was a significant difference in the incidence of recent postpartum complications between the two groups, the reasons for the analysis are as follows: the scar status can reflect the recovery effect of the last surgical incision to a certain extent, and patients with poor scar status have poor recovery from the previous cesarean section.

The ROC curve showed that the AUCs of abdominal scar score for predicting pelvic floor functional rehabilitation abnormalities, vaginal microecological abnormalities and complications were 0.806, 0.871, 0.844, The abdominal scar score can indicate that women have high risk of pelvic floor dysfunction, vaginal microecology and complications.

## Conclusions

The abdominal wall scar score has a certain effect on predicting pelvic floor function rehabilitation, vaginal microecology and complications after cesarean section, and it can help the medical staff to adjust the treatment measures in time, which can be used as an auxiliary means of preoperative examination. However, the sample size included in this study is too small, and the effectiveness of its clinical prediction needs further study.

## Supplemental Information

10.7717/peerj.16012/supp-1 Supplemental Information 1 Raw data.

## Additional Information and Declarations

### Data Availability

The following information was supplied regarding data availability: The raw data are available in the Supplemental Files.
